# Using Sub-Network Combinations to Scale Up an Enumeration Method for Determining the Network Structures of Biological Functions

**DOI:** 10.1371/journal.pone.0168214

**Published:** 2016-12-16

**Authors:** J. Y. Xi, Q. Ouyang

**Affiliations:** 1 Center for Quantitative Biology and Peking-Tsinghua Center for Life Sciences, Academy for Advanced Interdisciplinary Studies, Peking University, Beijing, China; 2 State Key Laboratory for Artificial Microstructures and Mesoscopic Physics, School of Physics, Peking University, Beijing, China; Queen's University Belfast, UNITED KINGDOM

## Abstract

Deduction of biological regulatory networks from their functions is one of the focus areas of systems biology. Among the different techniques used in this reverse-engineering task, one powerful method is to enumerate all candidate network structures to find suitable ones. However, this method is severely limited by calculation capability: due to the brute-force approach, it is infeasible for networks with large number of nodes to be studied using traditional enumeration method because of the combinatorial explosion. In this study, we propose a new reverse-engineering technique based on the enumerating method: sub-network combinations. First, a complex biological function is divided into several sub-functions. Next, the three-node-network enumerating method is applied to search for sub-networks that are able to realize each of the sub-functions. Finally, complex whole networks are constructed by enumerating all possible combinations of sub-networks. The optimal ones are selected and analyzed. To demonstrate the effectiveness of this new method, we used it to deduct the network structures of a Pavlovian-like function. The whole Pavlovian-like network was successfully constructed by combining robust sub-networks, and the results were analyzed. With sub-network combination, the complexity has been largely reduced. Our method also provides a functional modular view of biological systems.

## Introduction

In most well-studied biological experiments, biological functions are known, whereas the corresponding mechanisms are hidden. One of the central tasks in systems biology is to resolve the hidden logic underlying observed phenomena. Technologies such as micro-arrays and protein chips have been designed to fulfill these tasks [1]. In theory, a biological function can be considered as a result of the dynamic behavior of a regulatory network, in which proteins, genes, and other components are represented as nodes, and the regulatory mechanisms (such as phosphorylation and transcription) are represented as edges. For a set of interaction rules, the dynamics of the control systems can be simulated. If the candidate structures of networks can robustly perform the aim function, they can be selected and analyzed. This analysis would help experimentalists identify candidate network structures, and to understand the logic of the biological mechanism being determined. In this content, the relationships between network topologies and biological functions have been well studied [2–13].

In biological systems, molecular machines are hypothesized to have common features [14–16]. These observations may allow us to build a “Mendeleev-like” table relating basic biological functions and the corresponding control networks. For this purpose, Ma *et al*. [17] provided an enumeration method to search for simple networks that perform an adaptation biological function. In their work, the aim function and regulation type (enzymatic reaction interaction) were defined mathematically. All possible three-node networks were enumerated, and each one was evaluated using 10,000 sets of parameters with Latin Hypercube sampling [18]. Robustness was represented by the percentage of parameter sets that could perform the aim function (termed the Q-value), and topologies with higher Q-values were selected and analyzed. Their analytical result shows that there are only two basic network structures that can perform the adaptation function. Based on this innovative approach, function-network correspondence has been reported for other biological functions, such as dose-response alignment [19], semi-log dose response [20], self-organizing cell polarization [21], and Turing pattern formation [22].

The enumeration method tests every candidate network topology and uses Latin-cubic parameter sampling to search the whole parameter space, leading to global optimal solutions. However, a fatal shortcoming in this method is its high computational cost. For three-node networks, there are 19683 (**3**^9^) candidate topologies, and it takes approximately 3 days for a parallel program to complete the search. For a four-node case, there would be 43046721 (**3**^16^) candidate networks, and the calculation would take more than 10 years with current computation systems. Conversely, if this method is restricted to three-node networks, the complex functions that could be investigated are limited. As biological systems are complex, applying this method to further our understanding of biological regulatory mechanisms would be largely restricted.

An alternative, frequently used method is the evolutionary algorithm [23–26] which evolves a network topology based on how well it performs an aim function. The evolutionary method can easily be scaled up to complex functions and networks, but global optimization is compromised due to the reliance on initial values.

It has been hypothesized that biological systems have hierarchical structures both in regulatory networks and in functions [1][14][27–29]. If we view a basic biological function and its corresponding regulatory network as an “atom”, we speculate that a more complex function and its corresponding regulatory network may be considered as a “molecule”. Indeed, Ma et al. have demonstrated that the function of segment polarity in Drosophila melanogaster can be separated into sub-functions, where each sub-function relates to a set of sub-networks. A combination of sub-networks will likely provide a network that can perform the complex function [30]. In another study, Whitaker et al. carried out synthetic biological work on modularizing prokaryotic signal transduction to engineer robust control of the two-component phosphor-transfer system [31]. Guet *et al*. developed diverse synthetic biological functions through changes in binary circuits’ connectivity [32].

These studies provide examples of using modularization to study biological functions. Intrigued by these studies, we set out to develop a solution to scale up enumeration methods using sub-network combinations. The method includes three procedures: First, a complex biological function is separated into several sub-functions. Second, the enumeration method is applied to search for sub-networks that are able to realize each of the sub-functions. Finally, complex whole networks are constructed by enumerating all possible combinations of sub-networks, and the optimal ones are selected and analyzed. We tested this new method on a Pavlovian-like function.

## Methods

### 1. Function definition and separation

Pavlov’s dog is a famous example of classical conditioning reflection. In reality, the neural circuit in the canine brain that supports this conditioning reflection function is very complex; our task was to find a simple and robust control network that can mimic this function. In our work, the Pavlovian-like function was simply defined as the following: Before training, food alone causes the dog to salivate, but ringing a ring does not. Training involves providing the signals of food and the bell ringing together to the dog. After training, based on the memory state of the training, ringing alone could lead to salivation.

**Fig 1:**
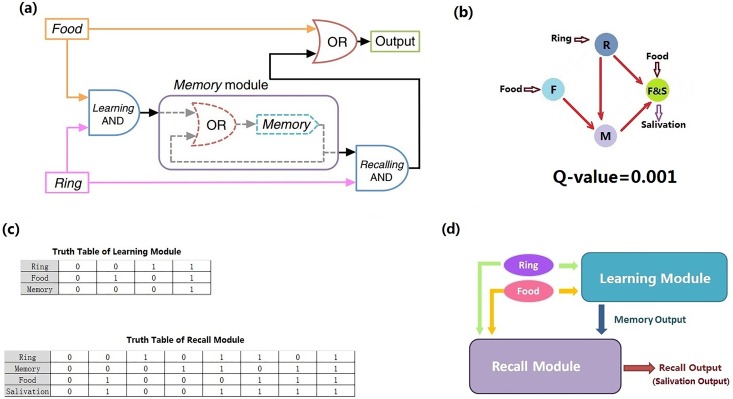
Pavlovian-like function. (a) Design of Pavlovian-like function using logic gates by Zhang *et al*. [33]. (b)The four-node network of Zhang *et al*. [33]. (c) Truth tables of learning and recall modules, respectively. (d) Our modularization of Pavlovian-like function. The learning and recall modules both receive the food and ring signals. Without memory signal, a food-only signal causes a high recall output (salivation), whereas the memory output remains low; a ring-only signal causes no response in both modules. When the food and ring signals occur together, the memory module can be turned on, and it remains in the ON state even after the signals are removed. After the memory output is triggered, the ring signal alone causes a high level of recall output.

Recently, Zhang *et al*. have constructed this Pavlovian-like conditioning system in *E*. *coli*, using a bottom-up synthetic biology approach [33]. In their work, the design of the regulation network was based on a model of Boolean logic, and genetic circuits were designed and synthesized following a digital logic design principle from electronic engineering. The control network consists of two AND gates, one OR gate, and one one-bit memory, with four control elements (nodes) altogether (Fig 1(a) and 1(b)). Although this simple logic design was successfully realized in the experiment, the robustness of the system was not considered; therefore, it might not be an optimal structure in term of robustness. In order to understand the design principles of natural network structure, one should go beyond Boolean logic.

The Pavlovian-like function can be divided into two sub-functions, or modules. The first sub-function (module) is the learning module, which receives the food and ring signals and generates the output of the memory state. Only when both food and bell ringing occur together, can the dog switch to a high-level memory state, and this memory remains a long time even after the food and ring signals disappear (Fig 1(d), learning module). The second sub-function (module) is the recall module, which receives the food, ring, and memory signals. Only when the memory and ring signals occur together, or there is a food signal, can the recall module generate a high state of salivation output (Fig 1(d), recall module). A proper combination of these two modules would generate the Pavlovian-like function (Fig 1(d)).

We tested and found that the Pavlovian-like function is too complex for a control network that has only three regulatory elements (nodes), thus the traditional enumeration method introduced by Ma *et al*. [9] cannot be applied to solve such a problem. This situation motivates us to seek sub-network combinations to scale up the enumeration method.

### 2. Sub-function network enumeration and analysis

**Fig 2:**
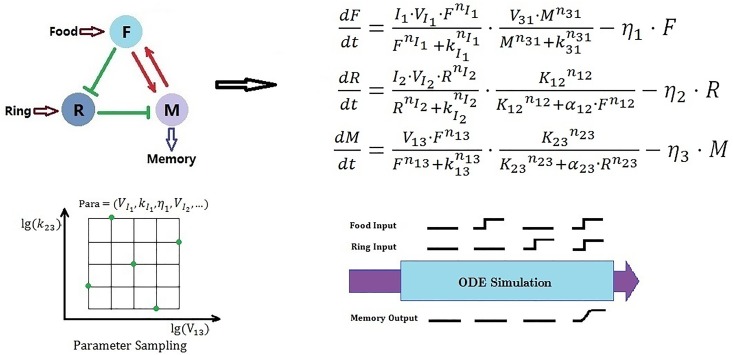
Illustration of the working procedure to search for a learning module, as an example.

According to the sub-network combination design (Fig 1(d)), after separating the Pavlovian-like function into sub-functions (a learning module and a recall module), we used the traditional enumeration method to select the robust sub-networks for each sub-function. Fig 2 provides a working procedure for searching for learning sub-networks. For each one of all possible three-node networks, we first established a set of Ordinary Differential Equations (ODEs) to describe the dynamic behavior of that network. We randomly selected a set of parameters to simulate the dynamic behavior of the sub-network, and used Latin hyper-cubic sampling to ensure that the parameters were selected uniformly in a log-scale parameter space. A large number of parameter sets (10,000) were sampled, and the network function was tested to see whether the network dynamics satisfy the aim function. For example, for the learning module, there are four types of input signals. A high output is achieved only when both food and ring signals are given, otherwise the output remains low. For a given network, if the set of parameters satisfied the aim function, it was selected and counted. Finally, when all the sets of parameters had been tested, we obtained the percentage of parameter sets that can perform the aim function (termed as Q-Value). If the Q-value of a network was larger than a properly set threshold, this network was considered robust and selected.

In order to compare the results of this study to the experimental work of Zhang et al. [33], transcription regulation was chosen as the node interaction rule. We followed the standard procedure to set ODEs according to the network [34][35]. In short, transcription regulation is represented as a Hill function. A positive regulation from node i to node j is written as:

$$V_{ij}\cdot H[A_{i},k_{ij},n_{ij}],$$

where H is the Hill function:

$$H[x,T,n]\equiv \frac{x^{n}}{x^{n}+T^{n}}.$$

Negative regulation from node i to node j is written as:

$$\frac{k_{ij}K_{ij}^{n_{ij}}}{K_{ij}^{n_{ij}}+\alpha_{ij}\cdot A_{i}^{n_{ij}}},$$

And self-degradation of each node i is written as:

$$-\eta_{i}\cdot A_{i},$$

where “A” is concentration of node i; “V” denotes the maximal production rate; “T” is the half-maximal concentration constant; “k” represents the inhibition; “n” is the hill coefficient; “K” means the inhibition coefficient, and “*α*”is the parameter determining the relative weight of inhibition. For the ranges of parameters, “V” in the formulas ranges from **10**^−3^ to **10**^2^, “k” ranges from **10**^−6^ to 1, “K” ranges from **10**^−3^ to 10, “*α*” ranges from 0 to 1, “*η*” ranges from **10**^−12^ to 1, and “n” ranges from 1 to 3.

Positive regulations from different nodes to one node are multiplied to form AND-logic-like continuous dynamics; negative regulations from different nodes to one node are additive to form OR-logic-like continuous dynamics. In addition, self-degradation exists for each node; it is proportional to the density of the node.

## Results

### 1. Learning sub-function

We defined the roles of three nodes in the learning module as follows: the first node (*R*) receives the input signal of the ring, the second node (*F*) receives the food signal, and the third node (*M*) acts as the output node for memory. We used the ODE solver tool in GSL to get the series of values of output density over a time period (10000 arbitrary unit), with the step length set to be **10**^−4^. Each time we got only an output-t curve under one set of parameters and one initial state. Based on the recorded curve, the program was designed to judge whether it came to a steady state. The steady state of an output in our manuscript is defined as: taking an arbitrary value of output in the last 1000 steps as “*y*”, and the average of the output values from t = 9000 to t = 10000 as “*y*_0_”, if they satisfy the relation: **|** *y* − *y*_0_ **|** < **0.01**⋅*y*_0_, where “| |” denotes absolute value, the output is considered as steady state. Different initial values might lead to different results even under the same set of parameters. The rule out the multiplicity states of outputs, for each simulation and nodes except for output nodes, we chose 10 different sets of initial values of nodes randomly from the set **[0, 50]**^*M*^ (M is the number of the nodes except for output nodes). We set the initial values of output nodes to 0.1 because they must be low at beginning. Only if for all initial conditions the output converge to the same steady state, the corresponding networks could be selected.

We used the ratio of the output value when the input signal is on over the output value when the input signal is off to judge the state of the output. The memory-off state was defined as the ratio was less than 5, and the memory-on state was defined when the ratio was higher than 15, these were considered as “0” and “1”, respectively, such that the output was switch-like. If the ratio was between 5 and 15, the result was not significantly switch-like; therefore, we excluded the networks whose output ratios in this region. The memory state requires the output value to remain high after we remove the input signals. This was tested as following: After the output value came to a platform, the input signals were removed and the simulation continued. At T = 50000, if the output value is not lower than **0.8***y*_0_, this system was considered as being able to perform the learning function. Using the enumeration method, we sampled all candidate of three-node networks (19683 in total), and networks with Q-values higher than 0.007 were selected as robust structures for the learning function.

**Fig 3:**
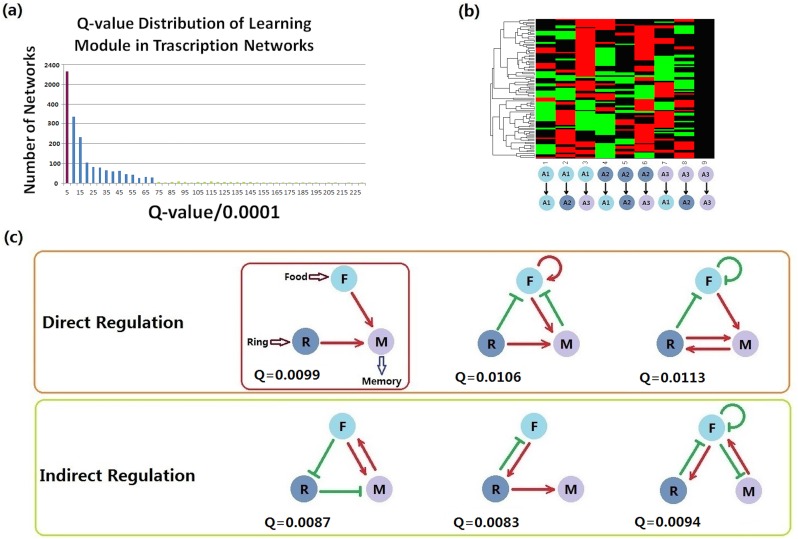
Network topologies that perform the learning function robustly. (a) Q-value distribution of the learning module in which networks with Q-values of 0 were neglected. The purple column shows that the Q-values of most networks were less than 0.0005. Networks with Q-values >0.007 are represented by green columns; the total number of such robust networks was 101. (b) Cluster results from the learning module in which the red areas illustrate positive regulation between the corresponding nodes, the green areas indicate negative regulation, and black indicates no regulation. (c) Core structures of the learning module. The red arrows represent positive regulation; the green edges with a bar on each end represent negative regulation. The method of searching for core structures was to choose clustered rows with same colors at the same positions with relatively large areas. The larger the area with the same color is, the greater the clustering of networks with the same regulation is.

Fig 3 provides the results from the three-node enumeration calculation for the learning module. Among the networks that could perform the function, only 101 had Q-values higher than 0.007(Fig 3(a)). For those robust networks, cluster analysis (Fig 3(b)) and visual inspection revealed some fundamental topologies (Fig 3(c)). The simplest structure for the learning module was a positive regulation from each of the input nodes (food and ring). This topology was among the networks with high Q-values, but was not the most robust one. Additional edges based on this simple structure provided extra robustness. These demonstrated that a robust learning module might have a more complex structure than the simplest logic design, which differs from the Boolean case. Besides the simplest direct structure, positive regulation from the input nodes to the memory node might be arranged indirectly (Fig 3(c), second row).

### 2. Recall sub-function

**Fig 4:**
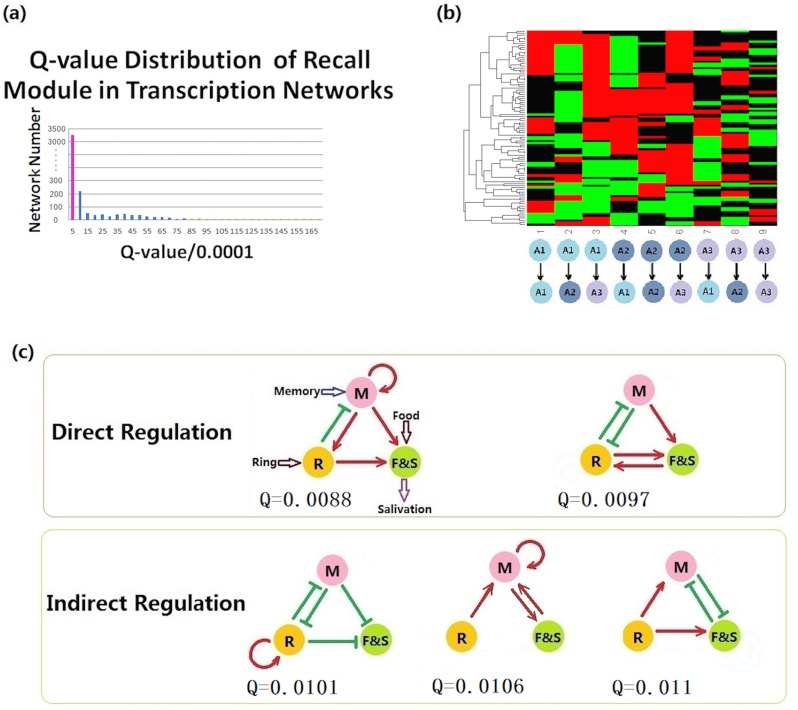
Network topologies that perform the recall function robustly. (a) Q-value distribution of recall module in which networks with Q-values > 0.008 are represented by green columns and the total number of such robust networks is 96. (b) Cluster result for recall module. (c) Core structures of the recall module.

Similar calculation procedures were conducted on the search for the recall module, the only difference being that the recall module had three input nodes: memory, ring, and food. Because we were limited by the three-node configuration, we combined the food signal (input) and salivation (output) together (F&S). Similar as in Learning module selection, the initial values of memory and ring nodes were chosen from [0, 50] randomly, while the initial value of output node (F&S) was set to be 0.1. We used the same thresholds of 5 and 15 for the on and off signals and the same criterion of “steady state”. Due to the function of Recall, food signal is added to the regulation on the output node, while the Ring signal and Memory signal are multiplied. The Q-value threshold for this network selection was set to be 0.008. Among all 19683 ($3^{3^{2}}$ networks, only 96 networks had Q-values higher than this threshold (Fig 4(a)).

The recall modules always consist of direct or indirect positive regulations from nodes *M* and *R* (which receive the memory state and ring signals) to node *F&S* (which acts as both the receiver of the food signal and the recall output). To ensure that the output level does not grow too high after receiving one of the memory and ring signals, there was, in some cases, negative regulation from one of the two receivers to the other, which then indirectly exerted positive regulation on node *F&S* (Fig 4(c)).

### 3. Combination rules and results

**Fig 5:**
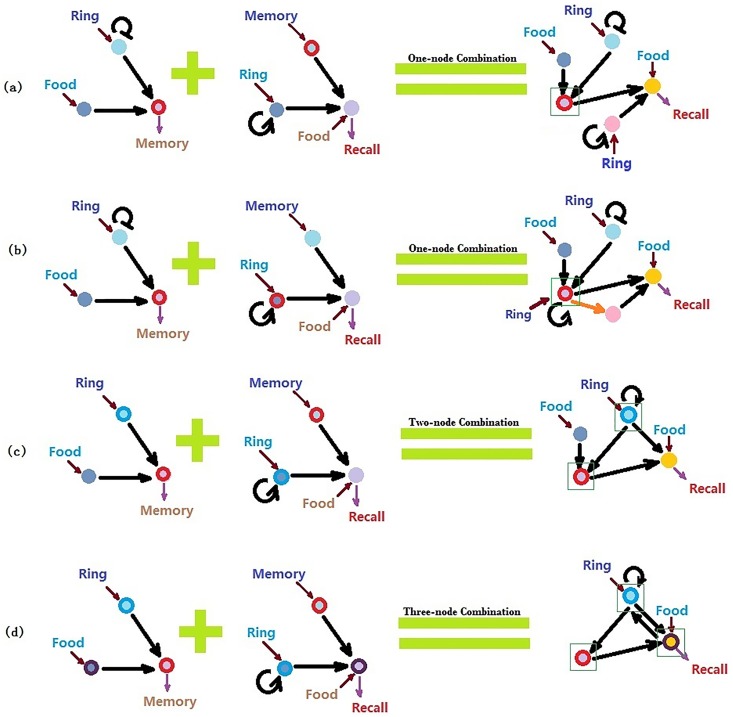
Examples of sub-network combinations. Circles of the same color are combined; green squares illustrate the combined nodes. (a) An example of a one-node combination: the memory nodes of two sub-networks are combined. (b) An example of the combination of the ring node of the recall module and the memory node of the learning module: positive regulation from the memory node of the learning module to the memory node of the recall module is added. (c) An example of a two-node combination: the *R* and *M* nodes of two sub-networks are combined. (d) Three-node combination: the *R*, *F*, *M* nodes of two sub-networks are combined.

The top 101 learning networks and the top 96 recall networks, ranked by their Q-values, were used as sub-network pool combinations in order to obtain networks for the Pavlovian-like function. For each combination, one sub-network was chosen from each pool. The combination rules can be grouped into three types: one-node combinations, two-node combinations, and three-node combinations. In the calculation, a combination was first tested under the logic concern that the regulation of the combined nodes must not be in contrast with each other. For example, if *F*_1_ had a positive regulation on itself, whereas *F*_2_ had a negative regulation on itself, the two sub-networks could not be combined. However, the combination was allowed if *F*_1_ had a positive regulation on itself, and *F*_2_ had no self-regulation. If nodes *F*_1_ and *F*_2_ were combined, and *R*_1_ and *R*_2_ were also combined, we would need to consider the logical correctness of both self-regulations and regulations from one node to the other. For example, if the regulation from *F*_1_ to *R*_1_ was positive, and the regulation from *F*_2_ to *R*_2_ was negative, then this combination would be logically impossible. In addition, the input and output nodes could not be in conflict with each other. The node that receives the food signal cannot also receive the ring signal, and the output node of the memory function cannot be the output node of the recall function (Fig 5).

We enumerated all logically possible one-node, two-node, and three-node combinations, and selected robust Pavlovian-like networks using Latin hyper-cubic parameter sampling. When the structures of the two modules are combined, the functions should be maintained, but one combined node may have multiple roles without conflict. A memory output node in learning module can be the input node in recall module, and if there is no direct combination of these two “memory” nodes, we added a positive regulation from the memory output node to the recall memory-input node. The rules of regulations and ranges of parameters were the same as in searching for learning and recall modules. The thresholds for the on and off signals of Memory and Recall outputs were 5 and 10, respectively. Each combined network must go through test of both the learning function and the recall function. During the selection of Pavlovian-like networks, different sets of Food signals and Ring signals were given to the food-input and ring-input nodes of both the learning module and recall module. The test of learning module and recall module was similar to the process of searching for the sub-networks alone. The only difference was that when the food signal was given to the food-input node of learning module, recall output must also be on. Our calculation results showed that the combinations heavily restrict the number of networks that can perform the aim function. Within 46624 logically possible one-node combination networks, only 59 networks could perform a Pavlovian-like function. Similarly, within 21055 logically possible two-node combination networks, only 36 networks could perform a Pavlovian-like function. For the three-node combination, no network could perform the Pavlovian-like function.

**Fig 6:**
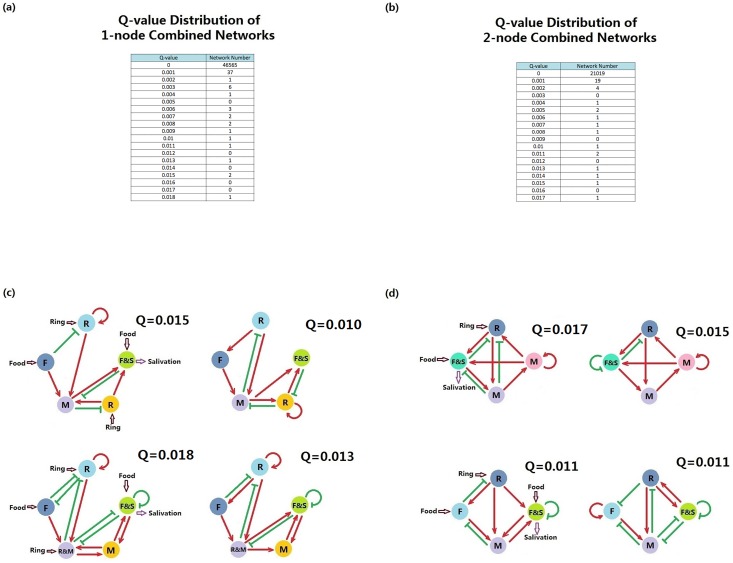
Results of sub-network combinations. (a) Q-value distribution of one-node combinations. (b) Q-value distribution of two-node combinations. (c) Samples results from one-node combinations. (d) Sample results from two-node combinations.

Fig 6 represents the results of these combinations. The Q-value distributions of the combinations are shown in Fig 6(a) and 6(b). Only a few networks can perform the Pavlovian-like function robustly. There are two types of one-node combinations: one is the combination of the output node of the learning module and the memory input node of the recall module, and the other is the combination of the output node of the learning module and the ring input node of the recall module. No other types of combinations were seen among the enumeration results. The two networks with the highest Q-values for each type are shown in Fig 6(c).

Two types of two-node combinations stand out in the enumeration: one is the combination of the ring and food nodes in each module, together with one positive regulation from the memory node of the learning module to the memory node of the recall module; the other is the combination of the memory node of the learning module and the memory node of the recall module, together with the combination of ring nodes in each module. The two networks with the highest Q-values of each type are shown in the Fig 6(d). Within the two-node combination results, the design of Zhang *et al*. [33] was also selected, but with a relatively low Q-value (0.001).

From the results of these combinations, we found that a Pavlovian-like network with high Q-values always requires high Q-values in its sub-networks (S1 Fig), consistent with Ma’s finding [30]. We also found that the combination of sub-networks may require minimal conflicts among regulations. One way to quantify this restriction is to calculate the regulation entropy [36]. To test if the regulation entropy is an important factor for the selection of networks, we chose 100 random five-node (corresponding to one-node combinations) networks and compared these with our selected networks. Our calculation results showed that for the random networks, the average regulation entropy is 0.7482, and the average regulation entropy of all logically possible combined networks is 0.2784, which demonstrates that the low regulation entropy is due to the requirement of logical correctness. The average regulation entropy of all the networks that perform a Pavlovian-like function is 0.2289, which is lower than the average regulation entropy of all possible combined networks, and much lower than the average regulation entropy of random five-node networks. This result indicates that sub-network combinations require minimal conflicts.

The situation is slightly different for four-nodes networks (corresponding to two-node combinations). In this case, for 100 random four-node networks the average regulation entropy is 0.5046, and the average regulation entropy of all logically possible combined networks is 0.1163. Conversely, the Pavlovian-like two-node combined networks have an average of regulation entropy of 0.3229, which is lower than the result from random networks, but higher than the result from all logically possible combined networks. This indicates that low regulation entropy is not the only factor that determines the success of sub-network combinations.

## Discussion and Conclusions

Compared to the traditional enumeration method, the complexity of the sub-network combination is largely reduced. For a complex function that consists of *n* nodes, the total number of all candidate networks (including all possible logically incorrect ones) for 3-node sub-network combinations satisfies (for details see *Supporting Information*):

$$C_{N}∼3^{9}\cdot [\frac{N}{3}]+\frac{33}{16}\cdot 8^{\left[\frac{N}{3}\right]-1}\cdot m^{\left[\frac{N}{3}\right]}{\left[([\frac{N}{3}])!\right]}^{4}.$$

Where “N” is the total number of node in a network; “m” is the average number of high-Q-value sub-networks selected through enumeration of each module. Comparing this computation complexity with the complexity of the traditional enumeration method ($3^{N^{2}}$), our the sub-network combination method largely reduces computation cost. The new method is much easier to solve for current-generation computers with limited computational ability. However, we should emphasize that there is a heavy trade-off for this new method. While the traditional enumeration method covers all the topological space, the combination method can cover only a small portion of it. For example, in this study, we only selected around 0.5% (around 100 out of 19683) sub-networks for sub-networks combination, this operation eliminates most of possible structures, which might omit the most robust network. On the other hand, we believe that this operation is justified by the observation that biological systems have hierarchical structures, and the combination of robust sub-networks will most probably to produce the more robust functional network [30].

The distribution of Q-values demonstrates the necessity of dynamic concern in designing network topologies for an aim function. When Zhang *et al*. [33] constructed their Pavlovian-like function in E.coli, they designed one of the simplest candidate networks with four nodes (Fig 1(b)). Our dynamics simulation results showed that the Q-value of this network is only 0.001, while the most robust network in our design has a Q-value of 0.017. This indicates that the digital logic design principle for electronic engineering may not be suitable for designing circuits in synthetic biology, where robustness has a high priority. Our new method is more effective in choosing robust network topologies to perform the aim function.

The most robust Pavlovian-like networks obtained by sub-network combination always had more edges than the simplest logic design, which implies that robust networks performing complex functions may require more edges to stabilize the function of each sub-module. A possible reason is that when two modules are combined, they might affect each other’s function. To cope with this complexity, the combined edges not only act as signal propagators, but also act as buffers for mutual interference of the sub-modules, which is consistent with the low regulation entropy.

The relatively low regulation entropy of sub-network combinations reflects the constraint on combination rules: no contradictions are allowed between each sub-module of the combined network. As the number of combined nodes increases, the difficulty of keeping this rule increases, thus there are fewer and fewer combined networks that can survive. Biological systems often obey the low-regulation-entropy rule [36], and our research on sub-network combination might be able to explain this phenomenon: some complex biological systems might be combined from simpler biological systems, and when they were combined (sharing certain common elements), both logically and dynamically, the combination required a low regulation entropy.

Because of the functional symmetry of Food and Ring input nodes in learning module (not in recall module), and the symmetry of Ring and Memory nodes in recall module, if the results are extremely biased to one side of the functional symmetric topologies, the whole parameter space cannot be represented by Latin-cubic sampling sufficiently. Our results have indeed shown symmetric topologies in the two modules. We did not exclude symmetric structures, future work can take the symmetry into account, and only consider testing one of the symmetric structure pairs. Applying symmetric consideration based on the functions people consider, the speed of calculating will be accelerated.

To ensure the generality of the sub-network combination, we also used this method on protein-protein interaction networks, and compared the results of protein interaction networks and transcription networks. The results are presented in *Supporting Information*. We demonstrated that sub-network combination shows similar advantages over traditional enumeration. Thus, sub-network combination may be a powerful method for designing network structures in synthetic biology. It ensures the robustness of a biological system in each step, and finally leads to much more robust solutions (high Q value) to a given aim function, compared with the network designed by Boolean logical model.

We note that this method can be expanded to networks with many more nodes and sub-modules. The success of the sub-network combination method not only largely reduces the high computational cost of the enumeration method, but also provides a new view on understanding biological systems: the modularization of biological functions.

Although it has several advantages, sub-network combination method is not perfect. As we stated before, while it is able to find a set of topologies with relatively high Q-value, it cannot guarantee the network with highest Q-value to be selected because we select only sub-networks with high Q-Value for combination. Moreover, in this research we only considered one of the interaction types, which is noncompetitive inhibition/activation. To make the method useful, further works may take different types of interactions between nodes into consideration, or different sub-networks follow different interaction types, and their combination can also follow different interaction type. At last, although our method decreases computational cost significantly, it still a NP-hard problem in calculation. The number of nots that the method can handle is still limited.

## Supporting Information

S1 Fig Q-value distribution of the results of 1-node and 2-node combinations. (TIFF)

S2 Fig The Comparison of Complexity of Traditional Enumeration and Sub-Networks Combination. (TIFF)

S3 Fig Results of the enumeration of learning modules in the protein-protein interaction case. (TIFF)

S4 Fig Results of the enumeration of learning modules in the protein-protein interaction case. (TIFF)

S5 Fig Q-value distributions of one-node and two-node combinations in protein interaction networks. (TIFF)

S6 Fig Comparison of high-Q-value one-node and two-node combination results of transcription regulation with the results from protein-protein interaction. (TIFF)

S7 Fig Examples of a Pavlovian-like network and a non-Pavlovian-like network. (TIFF)

S1 File Supporting File. (DOCX)
