# Supplementary figures and images for: Using Sub-Network Combinations to Scale Up an Enumeration Method for Determining the Network Structures of Biological Functions
Source: PLoS One. 2016 Dec 16;11(12):e0168214. doi: 10.1371/journal.pone.0168214 (PMC5161363; doi:10.1371/journal.pone.0168214)

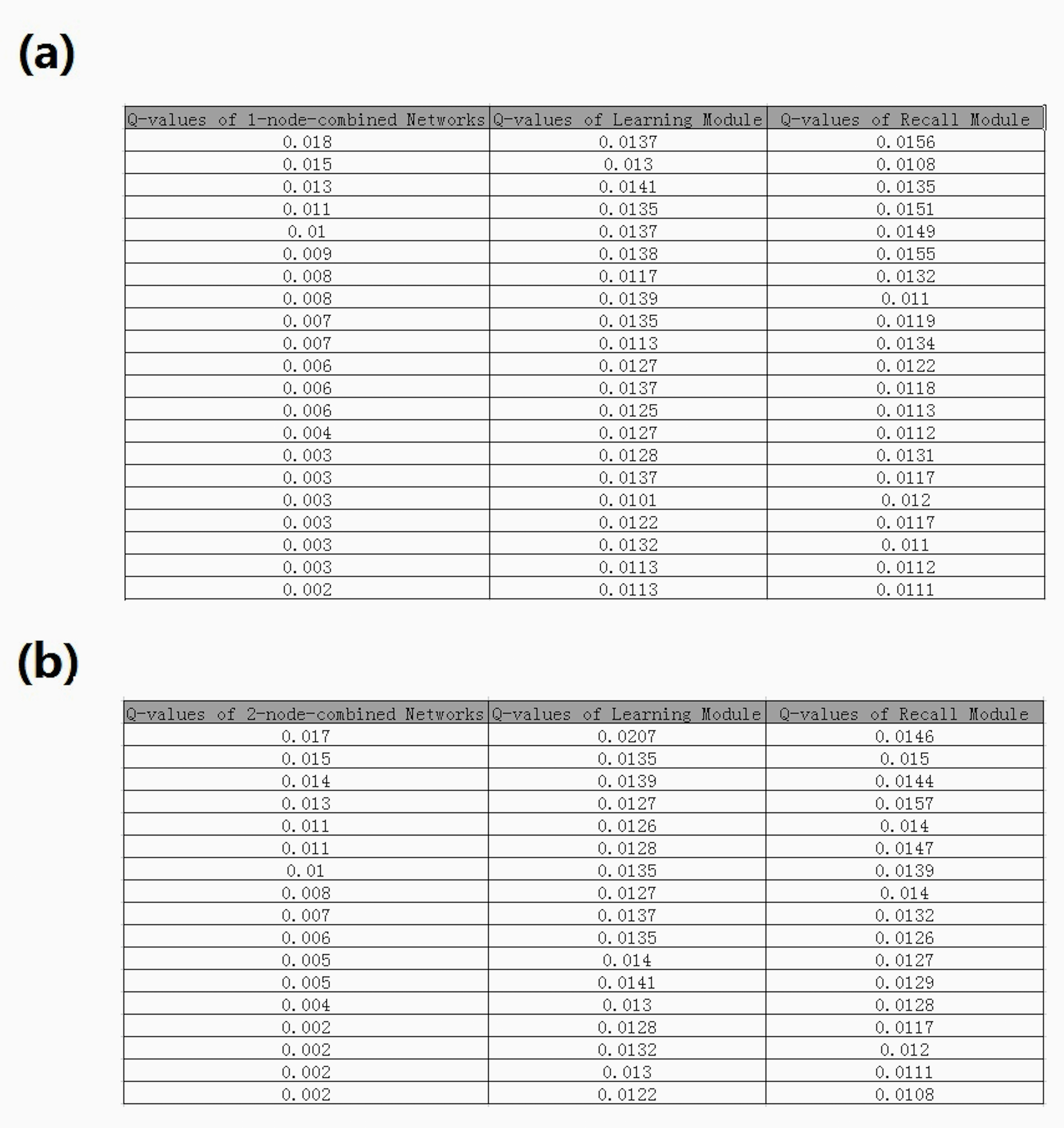

Supplement: S1 Fig — (TIFF) [file pone.0168214.s001.tiff]

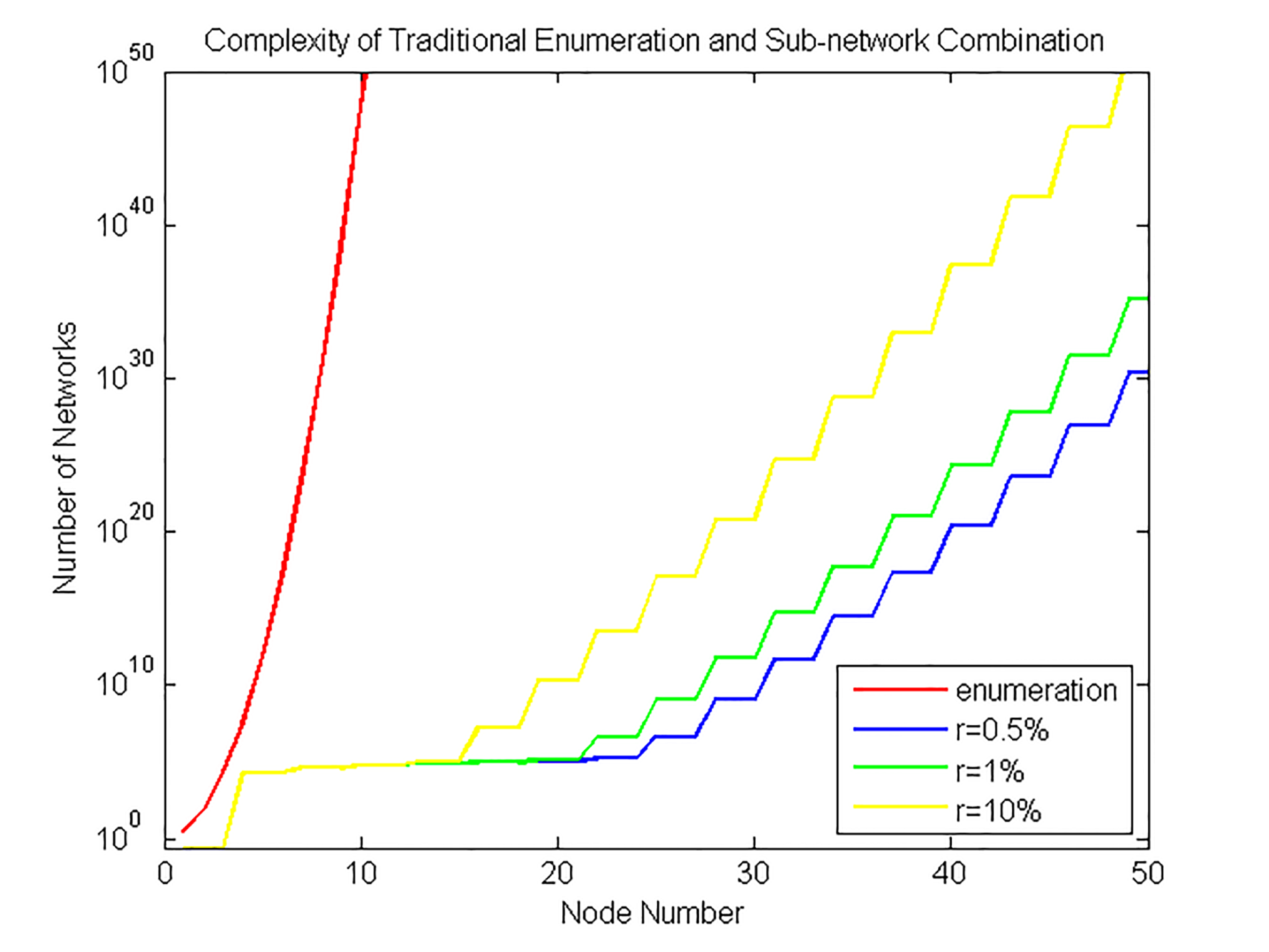

Supplement: S2 Fig — (TIFF) [file pone.0168214.s002.tiff]

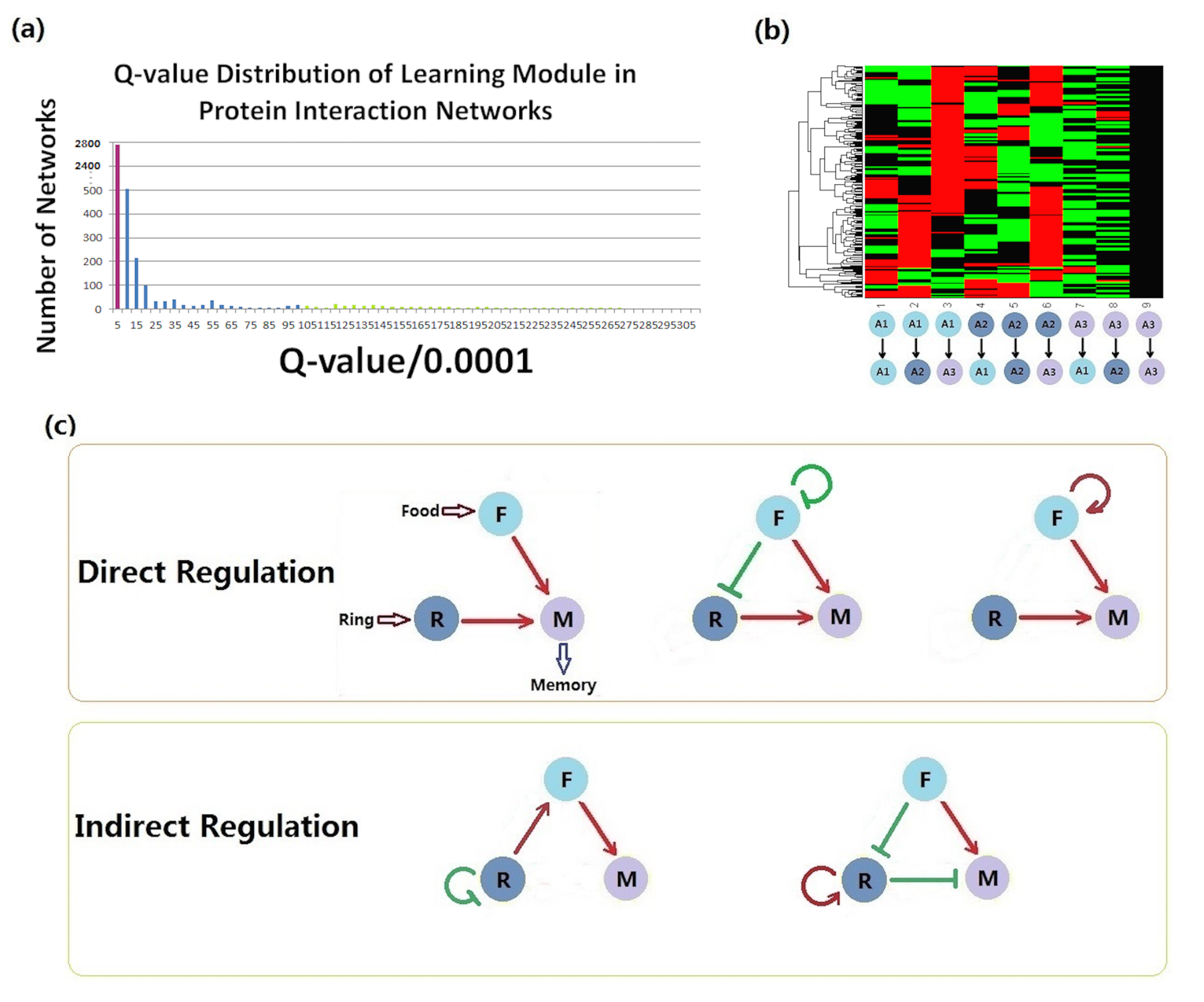

Supplement: S3 Fig — (TIFF) [file pone.0168214.s003.tiff]

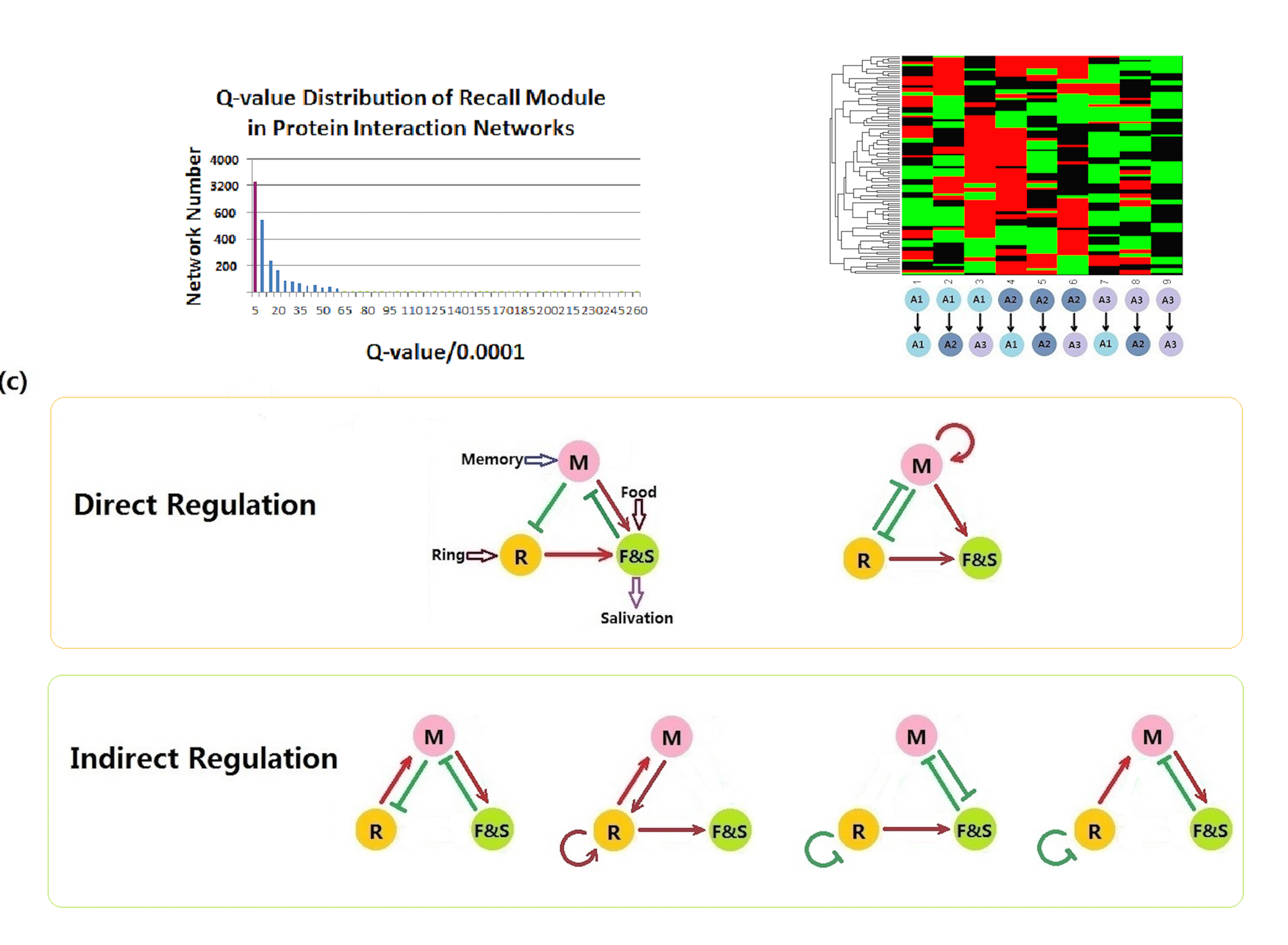

Supplement: S4 Fig — (TIFF) [file pone.0168214.s004.tiff]

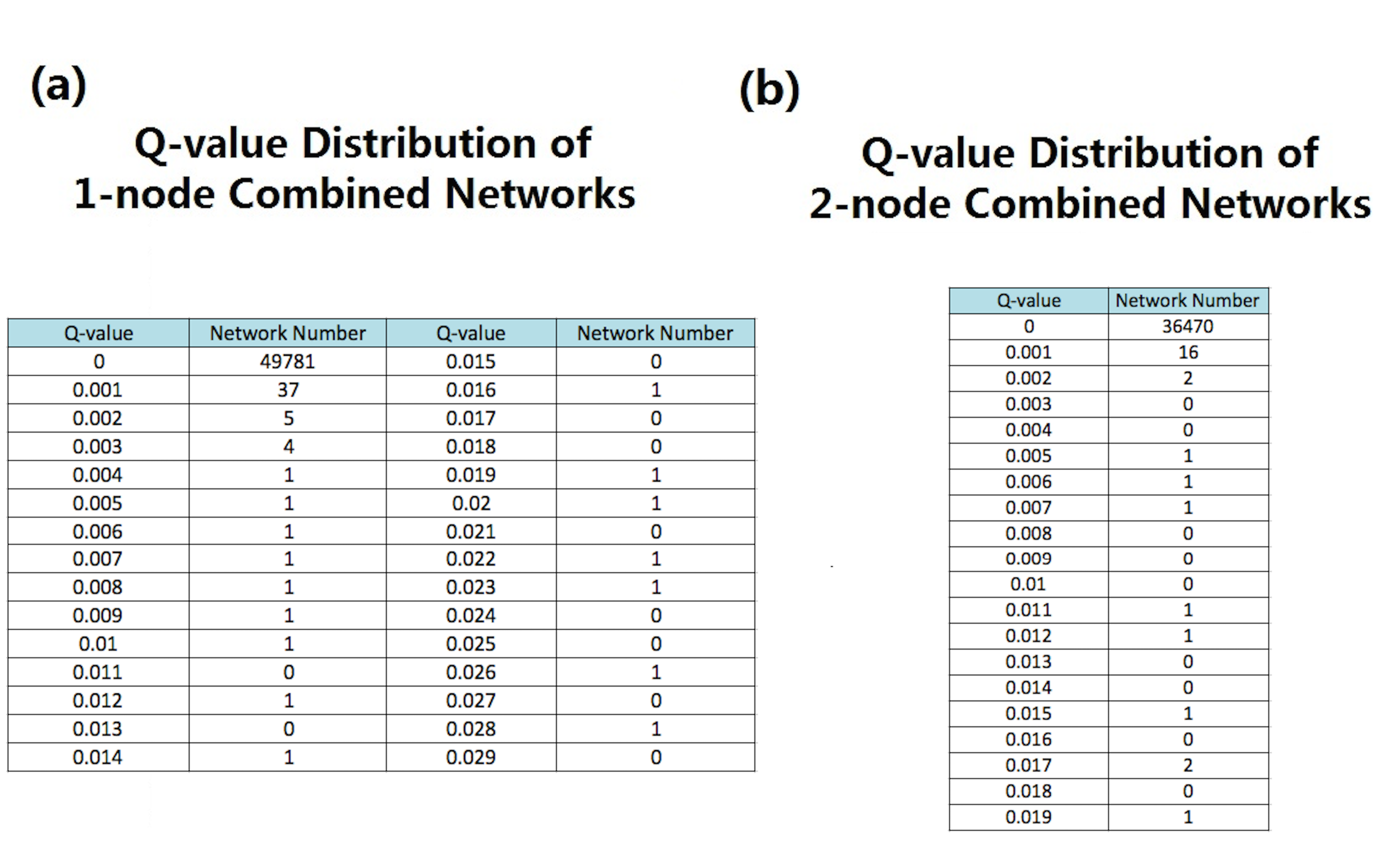

Supplement: S5 Fig — (TIFF) [file pone.0168214.s005.tiff]

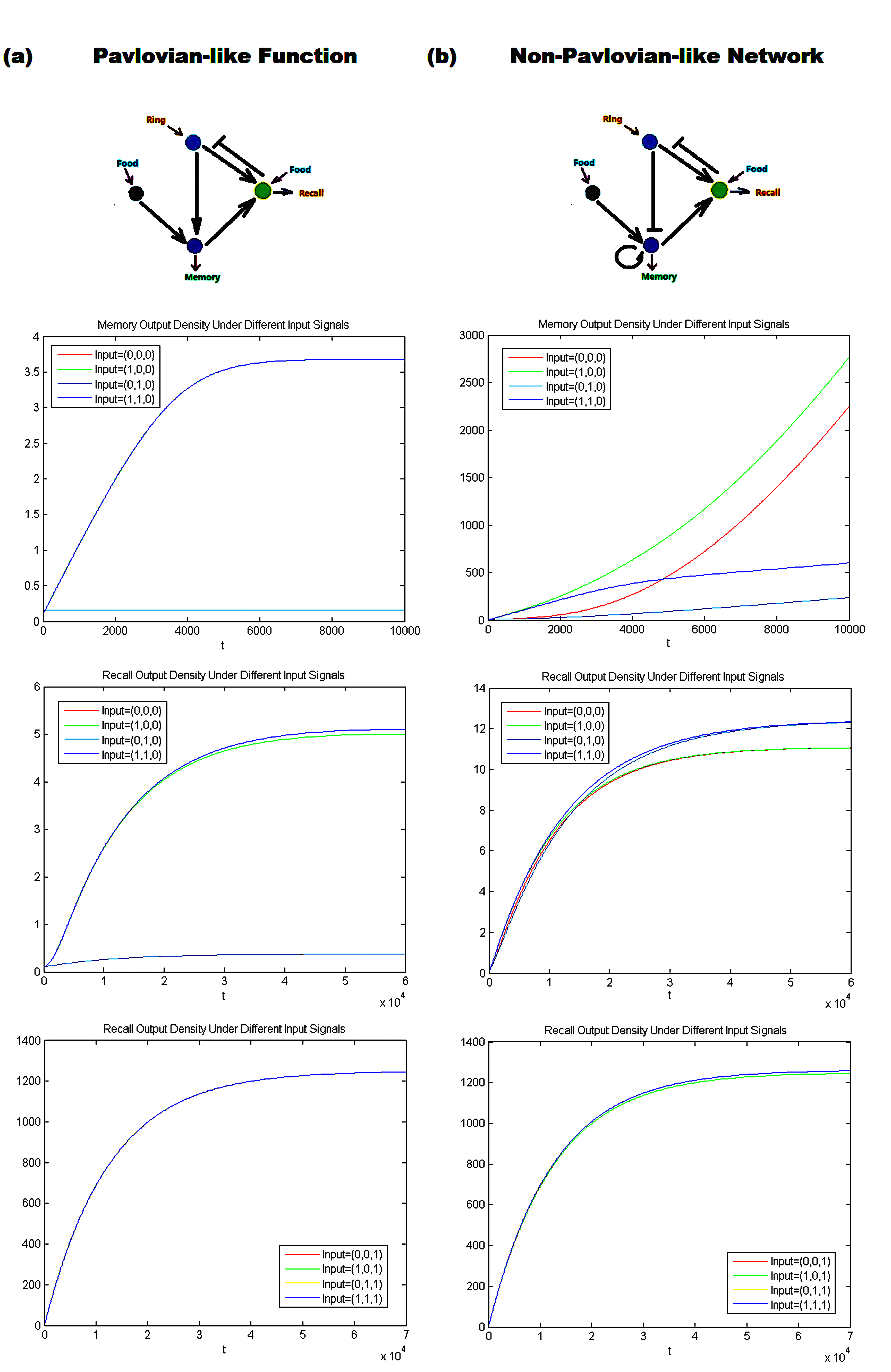

Supplement: S7 Fig — (TIFF) [file pone.0168214.s007.tiff]
