# Supplementary material for: Using Sub-Network Combinations to Scale Up an Enumeration Method for Determining the Network Structures of Biological Functions
Source: PLoS One. 2016 Dec 16;11(12):e0168214. doi: 10.1371/journal.pone.0168214 (PMC5161363; doi:10.1371/journal.pone.0168214)
Supplement: S1 File — (DOCX) [file pone.0168214.s008.docx]

**Supporting Information**

**1. Relationship between Q-Values of sub-networks and their combinations**

S1 Fig gives the Q-Values of combined networks and those of the sub-networks. These data indicate that the combined Pavlovian-like networks with high Q-values often require high Q-values for the corresponding sub-networks. This demonstrates that choosing sub-networks with high Q-values for the pool of combinations is reasonable.

**S1 Fig. Q-value distribution of the results of 1-node and 2-node combinations.**

**2. Complexity of sub-network combinations**

For a complex function which requires $n$ sub-modules, the total network number is:

$C_{n}=3^{9}\cdot n+m^{n}\cdot B_{n}$,

Where $3^{9}\cdot n$ is the total number of all three node sub-networks; and $B_{n}$ is the total number for the possible combinations for a given sub-networks combination; m is (High Q value). The $B_{n}$satisfies:

where $C_{j}^{i}$ is the combinatorial number, $A_{j}^{i}$ is the number of permutations. The recurrence in the first line means that a network with $k+1$ sub-modules can be constructed by combining one new three-node sub-network with one of all possible networks with k sub-networks. The term $C_{3}^{1}[A_{2k+1}^{1}+$ $A_{2k}^{1}+\cdot\cdot\cdot+A_{3}^{1}]$ inside the first “” means the number of possible one-node combinations of combining a new module to a complex network which consists k modules. To do the combination, we should firstly choose one node out of the three nodes of this new sub-network ($C_{3}^{1}$is the combinatorial number, and it means choosing one node from all the three nodes of this new module). Then we need to sample all possible combinations of this node to the given whole network with k sub-modules. The given whole network with k sub-modules may have $2k+1,2k,2k-1,...,3$nodes, so we must consider all of the possible cases, which is$A_{2k+1}^{1}+A_{2k}^{1}+...+A_{3}^{1}$. Then we shall consider the two-node combination case, thus the second term $C_{3}^{2}[A_{2k+1}^{2}+A_{2k}^{2}+...+A_{3}^{2}]$ in the first “”, in which the meaning of the permutations is that different orders of combinations to the same two nodes give different results. Similarly, we have the third term: $C_{3}^{3}[A_{2k+1}^{3}+A_{2k}^{3}+...+A_{3}^{3}]$ corresponds to the three-node combination. According to the basic mathematics, $A_{2k+1}^{1}+A_{2k}^{1}+...+A_{3}^{1}=$ $(k+2)(2k-1)$; $A_{2k+1}^{2}+A_{2k}^{2}+...+A_{3}^{2}=\frac{1}{3}(2k+1)(k+1)(4k+3)-(k+2)(2k+1)-5$; $A_{2k+1}^{3}+A_{2k}^{3}+...+A_{3}^{3}=(2k+1)(4k^{3}+2k^{2}-k)$ $.$Then after simple calculations we get $B_{k+1}<B_{k}(8k^{4}+16k^{3}+6k^{2}+6k-24)$. We can now get the inequality: $B_{k+1}<B_{k}(8k^{4}+16k^{3}+$ $6k^{2}+6k-24)<B_{k}\cdot8(k+1)^{4}$. Then from the recursive relations between each $B_{k}$and $B_{k+1}$, we can easily get the final inequality. Thus, the total number of all possible networks for N-node networks satisfies the equation:

$C_{N}\sim3^{9}\cdot[\frac{N}{3}]+\frac{33}{16}\cdot8^{[\frac{N}{3}]-1}\cdot m^{[\frac{N}{3}]}[([\frac{N}{3}])!]^{4}$,

in which “$[\frac{N}{3}]$”represents the largest positive integer that is not larger than $\frac{N}{3}$.

In contrast, in the enumeration method, the number of all possible network structures for N nodes is:

$D_{N}=3^{N^{2}}$.

We show S2 Fig the comparison of the computational complexity of two methods. In this log-scale diagram, the computational complexity of traditional enumeration method follows an exponential curve, while the computational complexity of sub-network combination method shows a semi-linear behavior.

**S2 Fig. The Comparison of Complexity of Traditional Enumeration and Sub-Networks Combination.** The red curve shows that the computational cost of traditional “brute-force” approach increases steeply as the node number increases. The other three represent the computational cost of sub-network combination. From this figure we can see that sub-network combination method largely reduces the computational cost. The ratio of the selected sub-networks from each sub-module “m” to all possible three-node sub-networks ($3^{3^{2}}=19683$) is defined as “r” in the figure. When r (10% (yellow), 1% (green), and 0.5% (blue)) decreases, the complexity of computation decreases at the same time.

**3. Protein-protein interaction networks**

To ensure the generality of the efficiency of the sub-network combination method, we also used protein-protein interaction networks to construct Pavlovian-like networks. A typical positive regulation from node *i* to node *j* is written as the following component [Supp1]:

$X_{i}\cdot\frac{V_{ij}\cdot(1-X_{j})^{n}}{(1-X_{j})^{n}+K_{ij}^{n}}$.

The negative regulation is written as:

$-X_{i}\cdot\frac{V_{ij}\cdot X_{j}^{n}}{X_{j}^{n}+K^{n}}$.

As in the study by Ma *et al.*, we assume that if a node is not activated or inhibited by any node, there will be an activation or inhibition from an alternative constant source. The results for the learning and recall modules are shown in S3 and S4 Figs.

**S3 Fig. Results of the enumeration of learning modules in the protein-protein interaction case.** (a) The Q-value distribution of learning modules: networks with Q-values of 0 were neglected. The green columns represent the most robust networks with Q-values higher than 0.01. (b) Cluster result of networks with high Q-values (Q>0.01). (c) Core structures of the learning module, from the cluster result.

**S4 Fig.** **Results of the enumeration of learning modules in the protein-protein interaction case.** (a) Q-value distribution of learning modules, in which networks with a Q-value of 0 were neglected. The green columns represent networks with Q-values higher than 0.006. (b) Cluster result of networks with high Q-values (Q>0.006). (c) Core structures of the learning module, from the cluster result.

For the learning function, using the enumerating method, we sampled all possible three-node networks, each with 10,000 sets of parameters, chosen by Latin hyper-cubic sampling. The Q-value threshold was 0.01, which means that topologies with Q-values higher than 0.01 were selected as robust topologies for the learning function. In the protein interaction networks, the core structures of the learning module also can be classified into two groups, which contain direct and indirect regulations from the input nodes to the output node, respectively. The structures with direct regulations all contain the simplest direct positive regulations from two input nodes (nodes *R* and *F*) to the output node (node *M*).

For the recall module, the function is defined similarly. The only difference is that it has three input nodes for memory, bell, and food, respectively. The ratio of output levels in situations where memory and bell signals come together, or the food signal is added to that of the situation in which there is no input, should be higher than 20. In other input conditions, the ratio should be from 0.01 to 5, and the Q-value threshold is 0.006. Results with Q-values higher than 0.006 were analysed, and the core structures could be classified into two groups: direct and indirect regulations from node *R* and node M to node *F&S*.

We chose the top-ranked 100 networks each from the learning and recall modules, respectively, and used the same sub-network combination method to combine them and select robust Pavlovian-like networks. Within 49843 logically possible one-node combination networks, only 62 networks performed a Pavlovian-like function. There are two types of one-node combinations: one is the combination of the output node of the learning module and the memory input node of the recall module; the other is the combination of the output node of the learning module and the bell input node of the recall module. There was no other type of combination among the enumeration results, just as in the situation with transcription networks.

For the two-node combination enumeration results, there were 36497 possible logical combinations, but only 27 of them could perform a Pavlovian-like function. There were also two types in general (as seen for transcription networks): one combining the output node of the learning module with the memory input node of the recall module, while combining the bell input node of each module; the other combining the bell input nodes and the food input nodes of the two modules, and linking the output node of the learning module with the memory input node of the recall module.

For three-node combinations, no network could perform the Pavlovian-like function, as for the transcription regulation networks.

Similarities in the Pavlovian-like function for transcription regulation and for protein-protein interactions (for one-node and two-node combinations) are shown in S5 Fig. From these results, it is evident that a network with a high Q-value in transcription regulation case may be robust in the protein-protein interaction case.

**S5 Fig**. **Q-value distributions of one-node and two-node combinations in protein interaction networks.**

**S6 Fig.** **Comparison of high-Q-value one-node and two-node combination results of transcription regulation with the results from protein-protein interaction.** The dark purple edges and the dark green edges are the common edges found in both cases; these demonstrate the similarities in the results for these two regulation types.

**4. Examples of Pavlovian-like and non-Pavlovian-like networks**

To show the function of combined networks, we present examples of a Pavlovian-like network and a non-Pavlovian-like network, respectively.

The first is a Pavlovian-like network. The network structure is represented in S7 Fig (a). The ODEs and parameters are shown below:

$\begin{matrix} \frac{dy_{1}}{dt}=\frac{k_{1}\cdot I_{1}\cdot y_{1}}{K_{1}+y_{1}}-\eta_{1}\cdot y_{1} \\ \frac{dy_{2}}{dt}=\frac{k_{2}\cdot I_{2}\cdot y_{2}}{K_{2}+y_{2}}+\frac{k_{42}}{K_{42}+y_{4}^{2}}-\eta_{2}\cdot y_{2} \\ \frac{dy_{3}}{dt}=\frac{k_{13}\cdot y_{1}}{K_{13}+y_{1}}\cdot\frac{k_{23}\cdot y_{2}}{K_{23}+y_{2}}-\eta_{3}\cdot y_{3} \\ \frac{dy_{4}}{dt}=\frac{k_{24}\cdot y_{2}}{K_{24}+y_{2}}\cdot\frac{k_{34}\cdot y_{3}}{K_{34}+y_{3}}+\frac{k_{4}\cdot I_{4}\cdot y_{4}}{K_{42}+y_{4}}-\eta_{4}\cdot y_{4} \end{matrix}$ $\begin{matrix} k_{1}=0.501,k_{2}=0.501,k_{13}=0.1,k_{23}=0.01,K_{1}=5,K_{2}=5.1,K_{13}=0.001,K_{23}=0.001\eta_{1}=0.1013,\eta_{2}=0.1013, \\ \eta_{3}=0.000001,k_{24}=0.001,k_{34}=1,K_{24}=0.005,K_{34}=5,k_{4}=0.1,K_{4}=0.01,\eta_{4}=0.00008,k_{42}=0.5,K_{42}=1. \end{matrix}$in which $y_{1}$is the density of the food-signal-input node, $y_{2}$is the density of the ring-signal-input node, $y_{3}$ is the density of the memory output node, and $y_{4}$ is the density of Recall-output node, it also receives the third input signal of food. In the ODEs,$I_{1}$, $I_{2}$ are the input signals of food and ring in learning module, respectively. While $I_{4}$ is the input signal of food in recall module. The value of the input signals can be 0 or 1.

**S7 Fig. Examples of a Pavlovian-like network and a non-Pavlovian-like network.**

The second is a non-Pavlovian-like network, with the same definition of each element in the ODEs. The network structure is shown in S7 Fig (b):

$$\begin{matrix} \frac{dy_{1}}{dt}=\frac{k_{1}\cdot I_{1}\cdot y_{1}}{K_{1}+y_{1}}-\eta_{1}\cdot y_{1} \\ \frac{dy_{2}}{dt}=\frac{k_{2}\cdot I_{2}\cdot y_{2}}{K_{2}+y_{2}}+\frac{K_{42}}{K_{42}+y_{4}^{2}}-\eta_{2}\cdot y_{2} \\ \frac{dy_{3}}{dt}=\frac{k_{13}\cdot y_{1}}{K_{13}+y_{1}}\cdot\frac{k_{33}\cdot y_{3}}{K_{33}+y_{3}}+\frac{K_{23}}{K_{23}+y_{2}}-\eta_{3}\cdot y_{3} \\ \frac{dy_{4}}{dt}=\frac{k_{24}\cdot y_{2}}{K_{24}+y_{2}}\cdot\frac{k_{34}\cdot y_{3}}{K_{34}+y_{3}}+\frac{k_{4}\cdot I_{4}\cdot y_{4}}{K_{42}+y_{4}}-\eta_{4}\cdot y_{4} \end{matrix}$$

$$\begin{matrix} k_{1}=0.501,k_{2}=0.8,k_{13}=0.1,K_{23}=0.1,K_{1}=5,K_{2}=5.1,K_{13}=0.001, \\ \eta_{1}=0.1013,\eta_{2}=0.1013,\eta_{3}=0.000001,k_{24}=0.001,k_{34}=1,K_{24}=0.005, \\ K_{34}=5,k_{4}=0.1,K_{4}=0.01,\eta_{4}=0.00008,K_{42}=0.5,k_{33}=1,K_{33}=0.1. \end{matrix}$$

**5. Why do we choose a Pavlovian-like function with more than three nodes?**

In fact, if we omit the “one-node-one-input” requirement, the Pavlovian-like function can also be performed using a two-node network:

$$\frac{dx_{1}}{dt}=\frac{F}{0.5+F}+\frac{R^{2}}{0.5^{2}+R^{2}}\cdot\frac{x_{2}^{2}}{0.5^{2}+x_{2}^{2}}-0.1\cdot x_{1}$$

$$\frac{dx_{2}}{dt}=\frac{F^{2}}{0.5^{2}+F^{2}}\cdot\frac{R^{2}}{1^{2}+R^{2}}+\frac{0.4\cdot x_{2}^{4}}{2^{4}+x_{2}^{4}}-0.1\cdot x_{2}$$

with the output being $x_{1}$, the memory being $x_{2}$, F the food input, R the ring input.

However, there are still several reasons for us to study networks with more nodes: The biological reason for this requirement is the applicability in biological systems. In our model, each signal should be received by a node in the network. The nodes act as the interfaces, thus the selected network can be embedded into a larger system more easily. This property makes it convenient in experimental application. The application reason is that the main aim of our research is to develop a new method for systematically studying larger scale network reverse engineering problem in the spirit of enumeration method, Pavlovian-like function is just an example. More complex function can be solved in the same way. There is also a practical reason: Pavlovian-like function is a function which can be clearly modularized functionally, and it has also been studied experimentally, designed by a Boolean logic method. So it is suitable for testing our method.

[Supp1] Ma W, Trusina A, El-Samad H, Lim WA, Tang C. Defining network topologies that can achieve biochemical adaptation. Cell. 2009 Aug 21;138(4):760-73

**Supporting Information Captions**

**S1 Fig. Q-value distribution of the results of 1-node and 2-node combinations.**

**S2 Fig. The Comparison of Complexity of Traditional Enumeration and Sub-Networks Combination.**

**S3 Fig. Results of the enumeration of learning modules in the protein-protein interaction case.**

**S4 Fig.** **Results of the enumeration of learning modules in the protein-protein interaction case.**

**S5 Fig**. **Q-value distributions of one-node and two-node combinations in protein interaction networks.**

**S6 Fig.** **Comparison of high-Q-value one-node and two-node combination results of transcription regulation with the results from protein-protein interaction.**

**S7 Fig. Examples of a Pavlovian-like network and a non-Pavlovian-like network.**
